# Methane dynamics from a mixed plantation of north China: Observation using closed-path eddy covariance method

**DOI:** 10.3389/fpls.2022.1040303

**Published:** 2023-01-11

**Authors:** Wenwen Yuan, Hui Huang, Jinsong Zhang, Ping Meng, Jun Li, Tonggui Wu, Fang Zhou, Qingmei Pan

**Affiliations:** ^1^ Research Institute of Subtropical Forestry, Chinese Academy of Forestry, Hangzhou, China; ^2^ Key Laboratory of Tree Breeding and Cultivation, National Forestry and Grassland Administration, Research Institute of Forestry, Chinese Academy of Forestry, Beijing, China; ^3^ Collaborative Innovation Center of Sustainable Forestry in Southern China, Nanjing Forest University, Nanjing, Jiangsu, China; ^4^ Henan Xiaolangdi Earth Critical Zone National Research Station on the Middle Yellow River, Jiyuan, China; ^5^ Key Laboratory of Water Cycle and Related Land Surface Processes, Institute of Geographic Sciences and Natural Resources Research, Chinese Academy of Sciences, Beijing, China

**Keywords:** CH_4_ flux, closed-path eddy covariance, a warm-temperate mixed plantation, footprint, dynamic

## Abstract

Although an important greenhouse gas, methane flux in hilly forest ecosystems remains unclear. By using closed-path eddy covariance systems, methane flux was measured continuously from 2017 to 2019 in a mixed plantation in the Xiaolangdi area of the Yellow River in North China. The methane flux footprint and its diurnal and monthly variations were analysed, and its characteristics on rainy days are discussed. The results showed that: (a) the observation data were reliable with good spatial representation (b) The methane flux in the mixed plantation ecosystem had obvious diurnal and seasonal variations: the monthly average diurnal variation of the methane flux had a single-peak; the methane flux value was source in the daytime and sink at night. The daily mean maximum value of methane flux in growing season was lower than that in non-growing season with the maximum value appearing in March, and the minimum value in October. (c) The forest is an atmospheric CH_4_ source with the annual emission in 2017 of (3.31 g C·m^-2^·year ^-1^) >2019 (2.94 g C·m^-2^·year^-1^) >2018 (2.81 g C·m^-2^·year ^-1^), and the main influencing factor was precipitation. Rainfall affected CH_4_ flux with a lag period of approximately three days. Rainfall also changed the balance of CH_4_ flux between sink or source according to precipitation intensity and frequency.

## 1 Introduction

CH_4_ is the third largest greenhouse gas after water vapour and CO_2_, but its greenhouse effect over 100 years is 28 times that of CO_2_ (IPCC, 2013). On the global scale, approximately 10 Tg·a^-1^ of CH_4_ emissions are from unexplained sources (Megonigal and Guenther, 2008). The attribution of these emissions to particular sources and sinks is still an unresolved issue for the scientific community. Forest ecosystems cover most continental regions, and any sink-to-source transitions could have a non-negligible impact on global atmospheric CH_4_ budgets. Therefore, it is important to understand CH_4_ dynamics in forest ecosystems and CH_4_ exchange between the atmosphere and forests.

Considering that a major portion of forest soil is water-unsaturated, forests are generally assumed to be an insignificant atmospheric CH_4_ sink, representing about 6% of the global sink (Machacova et al, 2016). However, studies have revealed that forest ecosystems are not always CH_4_ sinks. Some global inversions of CH_4_ have indicated that broadleaf evergreen forests and tropical forests might be important CH_4_ sources (Frankenberg et al., 2005), contrary to the traditional view that forests always absorb atmospheric CH_4_. These results suggest that CH_4_ originates from a wider range of sources than previously considered. All biological surfaces in a forest, including living and dead wood, can exchange CH_4_, usually emitting CH_4_ (Covey and Megonigal, 2018; Pitz and Megonigal, 2017), indicative of a reduced sink and even a CH_4_ source in forest ecosystems. It is important to quantify CH_4_ fluxes in forest ecosystems (Shoemaker et al., 2014; Ueyama et al., 2018).

Ecosystem scale CH_4_ sources and sinks are uncertain in different forests. Smeets et al. (2009) revealed the Ponderosa pine forest ecosystem was a CH_4_ sink, while Miyama et al. (2010) had observed no CH_4_ flux changes from an oak-holly mixed forest in the warm temperate zone. Spatial heterogeneity of CH_4_ sources and sinks exist in the forest ecosystem, and while the forest is a CH_4_ source at the canopy scale, the forest ground surface absorbs CH_4_ (Simpson et al., 1999; Miyama et al., 2010; Mikkelsen et al., 2012; CoveyMegonigal, 2018; Nakai et al., 2020). The diurnal dynamic reflects a CH_4_ sink at the annual scale and in the growth season but indicates a CH_4_ sink during daytime and in the non-growth season (Ueyama et al., 2013; Sundqvist et al., 2015; Gao et al., 2016). Consequently, the underlying surface is complex due to diverse climates and forest types, consequently the aforementioned studies lack generalizability. In contrast to CO_2_ fluxes, there are relatively few studies on CH_4_ fluxes in forest ecosystems, and the published papers usually have relatively short durations of observation and data collection, making it impossible to evaluate and predict the global forest CH_4_ budget accurately, and the source-sink transition pattern and its role in the CH_4_ cycle are still not well understood.

Eddy covariance technology provides a reliable approach to measuring the CH_4_ fluxes. This study focuses on the methane flux dynamics at daily, seasonal, and interannual time scales based on 3 years (2017–2019) of eddy covariance flux observation in a mixed plantation of north China. The specific objectives of this study are to (1) identify CH_4_ dynamics at different time scales, and (2) clarify CH_4_ changes during the rainy days and provide a scientific foundation for accurately estimating CH_4_ fluxes.

## 2 Materials and methods

### 2.1 Site description

This experiment was conducted at the Xiaolangdi forest research station, Chinese Academy of Forestry Sciences. The station is located at Jiyuan County, Henan, China (35°01′ N, 112°28′ E; elevation 410 m), south of Taihang mountain and north of the Yellow River Basin. It has a warm-temperate continental monsoon climate. The average annual air temperature was 13.4°C. The annual mean rainfall was 642 mm, with an average growing season (April–October) rainfall of approximately 438 mm, making up approximately 60% of the whole year. The average annual sunshine hours are 2377.7 h. The stand is dominated by cork oak (*Quercus variabilis Blume*), which was planted in 1973, with a mean height of 10.5 m. The other trees include black locust (*Robinia pseudoacacia* L.) and arborvitae (*Platycladus orientalis*), with ages of 28 and 30 years and heights of 9.3 m and 8.2 m, respectively. The planting density was 1905 stems ha^-1^, and the stand coverage was 96%. The understory is sparse and mainly composed of sour jujube [*Ziziphus jujuba* Mill. var. *inermis* (Bunge) Rehd.], Bunge’s hackberry (*Celtis bungeana* BI), and green bristlegrass [*Setaria viridis* (L.) Beauv.]. The soil was principally brown loam, with an average thickness of 0.4 m. The flux observation tower (36 m) is situated at the centre of a large plantation area (7210 ha). The mean LAI of the mixed plantation is 6.3 during the growing season. The mean slope around the flux observation tower is 14° (Tong et al., 2012).

### 2.2 Methane flux and microclimate measurements

The closed-path eddy covariance (CPEC) system consists of a 3-D sonic anemometer (Model CSAT3; Campbell Scientific), a closed-path fast greenhouse gas analyser (FGGA; Los Gatos Research, Mountain View, CA, USA) and a dry vacuum scroll pump (XDS35i; BOC Edwards, Crawley, UK), requiring 520 W of power. All instruments were installed at a height of 30 m. Raw data were collected at 10 Hz and recorded by a CR5000 datalogger (Campbell Scientific).

In the CPEC system, an inlet tube situated at the same height as the anemometer, with a separation of 15 cm, was used to draw air into the FGGA. This analyser measures CH_4_, CO_2_ and H_2_O concentrations by off-axis integrated cavity ringdown spectroscopy (Baer et al., 2002). During the experiment, the pump drew the sample air through a 40-m tube (inner diameter: 5 mm) at flow rates of about 40 L min^−1^ into the measuring cell under an operating pressure of approximately 19 kPa. The air passed through an initial filter with a pore size of 100 μm to prevent dust and insects from entering the system, as well as through 5 μm and 2 μm external plum sharp filters at the end of the tube, and finally through two 2 μm metal filters (one internal and one external) before entering the measuring cell. The 100 μm filter is replaced every 6 months, while the external plum sharp filters and porous filters are replaced every 3 months to maintain clean optics and avoid inflow restrictions. Because the pump and the gas analyser have a high power requirement, the CPEC system ran on AC power during the measurement period.

### 2.3 Data processing

Processing of the raw EC data was performed using EddyPro 6.2 (LI-COR; available at www.licor.com/eddypro ). De-spiking and absolute limit determinations were included in the preliminary processing of raw signals (Vickers and Mahrt, 1997). At this preliminary stage, outliers also were discarded. This involves filtering for spikes and linear detrending. Double coordinate rotations were performed to align the mean vertical velocity measurements normal to the mean wind streamlines before carrying out scalar flux calculations (Wilczak et al., 2001). Using the covariance maximisation method (relative to the vertical velocity of temperature), the time lag was determined for each 30-min period. Half-hourly fluxes of CH_4_ were calculated as the mean covariance of vertical wind velocity and scalar fluctuations in CH_4_ concentrations. The Webb–Pearman–Leuning correction for density fluctuations arising from variations in water vapour was applied as described in Ibrom et al. (2007b). Low-pass filtering effects were assessed and corrected using the method of Ibrom et al. (2007a), based on *in situ* determination of water vapour attenuation and a model for the corresponding spectral correction factor. Quality control criteria according to Mauder and Foken (2004) were used to reject abnormal data. In addition, data were excluded when the pump stopped working, during maintenance or high temperature in summer, or when the sonic anemometer signal was degraded during heavy rain. The data from June to September in 2017 were used to determine the friction velocity (u*) threshold using R package ‘REddyProc’ (https://github.com/bgctw/REddyProc ). The average flux increased along with u* until it tended to level off and be independent of u* at around 0.1 m s^-1^. Data collected during weak turbulence were removed from analyses by filtering out all half-hourly flux measurements with a friction velocity (u*) below 0.1 m s^-1^. Time delays were calculated through the use of a cross-correlation function of the scalar fluctuation and the vertical wind velocity. The lag time was set as 8 s by comparing with an open-path eddy covariance system (Yuan et al., 2019). Atmospheric stability has a direct impact on the distribution of flux footprint, which is subsequently affected by wind speed, atmospheric temperature, and properties of the underlying surface. Therefore, the atmosphere was divided into stable state (Zm/L>0) and unstable state (Zm<0) according to the atmospheric stability Zm/L, where Zm is the height observed by instrument and L is the Obukhov length. The footprint model was used to analyse the source distribution of the flux signal. The data separated by more than three times the variance from the average were regarded as abnormal. Moreover, abnormal data reflecting instrument malfunction or unfavourable meteorological conditions (rain and dew) were also eliminated. A linear method was used to fill the gaps when data were missing, within 2 h. The larger gaps in the daytime and night-time were filled using the mean diurnal variation (MDV) and nonlinear regression methods, respectively (Falge et al., 2001). The finial calculation of CH_4_ fluxes value is positive which represent the methane source, and the value is negative with methane sink.

## 3 Result

### 3.1. Characteristics of CH_4_ flux footprint in Ecosystem

In this study, the eddy covariance flux data between 2017 and 2019 were analysed. A contour map of the flux footprint was drawn using Footprint software, taking the 90% flux footprint as the measurement target. The area was calculated by the grid area method and was used to analyse the changes of CH_4_ flux footprint.

**Figure 1:**
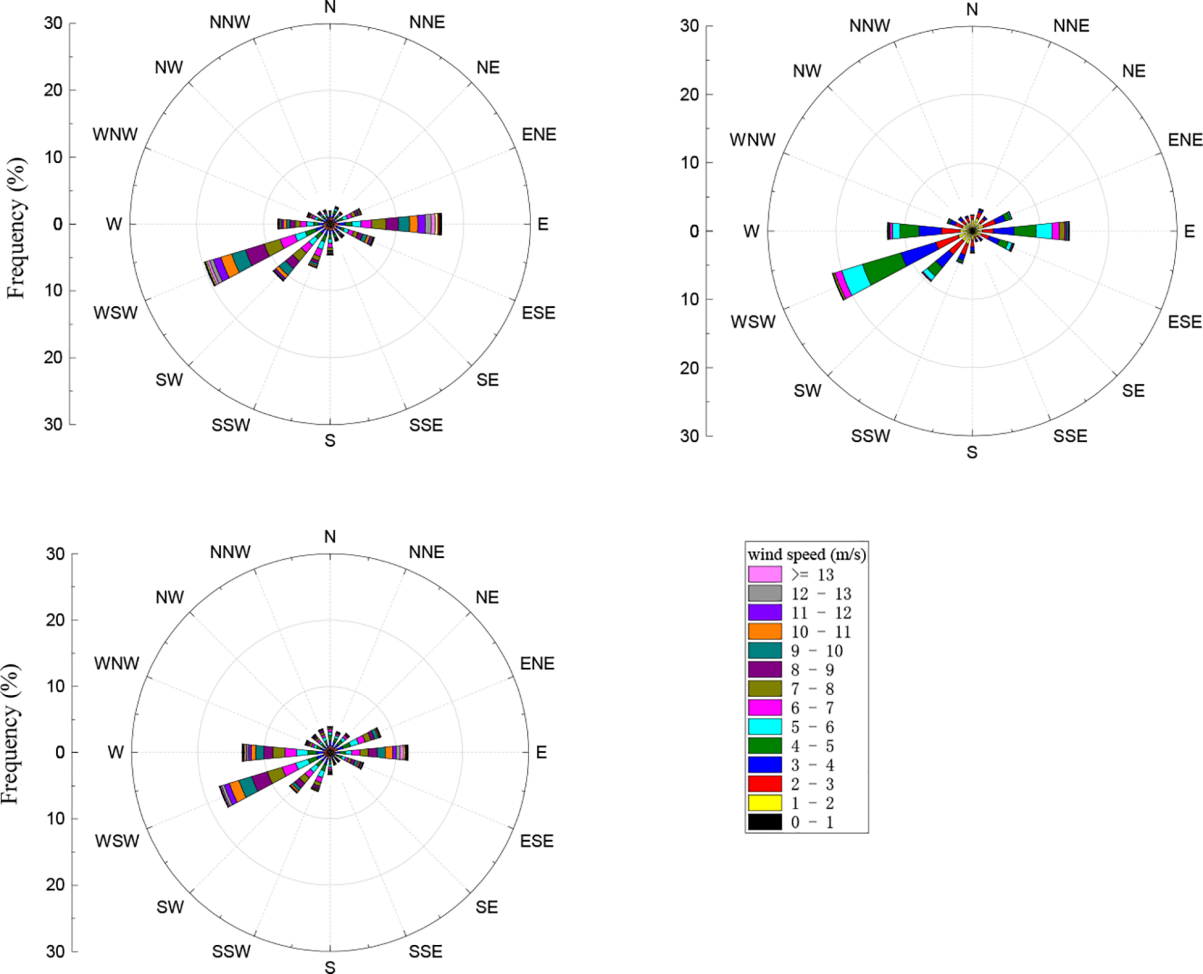
2017–2019 wind roses.

Analyses of the wind direction and speed data between 2017 and 2019 showed that the measurement area experienced easterly wind (at 90°) and west-south-westerly wind (between 225° and 270°) (**Figure 1**). This is consistent with the measurements obtained from previous years, which showed that winds in this area were predominantly east-north-easterly and west-south-westerly (Zheng et al, 2010). The maximum wind speeds in 2017, 2018, and 2019 were respectively 10.8 m·s^-1^, 12.3 m·s^-1^, and 10.6 m·s^-1^, while the average wind speeds were respectively 3.2 m·s^-1^, 3.2 m·s^-1^, and 3.1 m·s^-1^.

**Figure 2:**
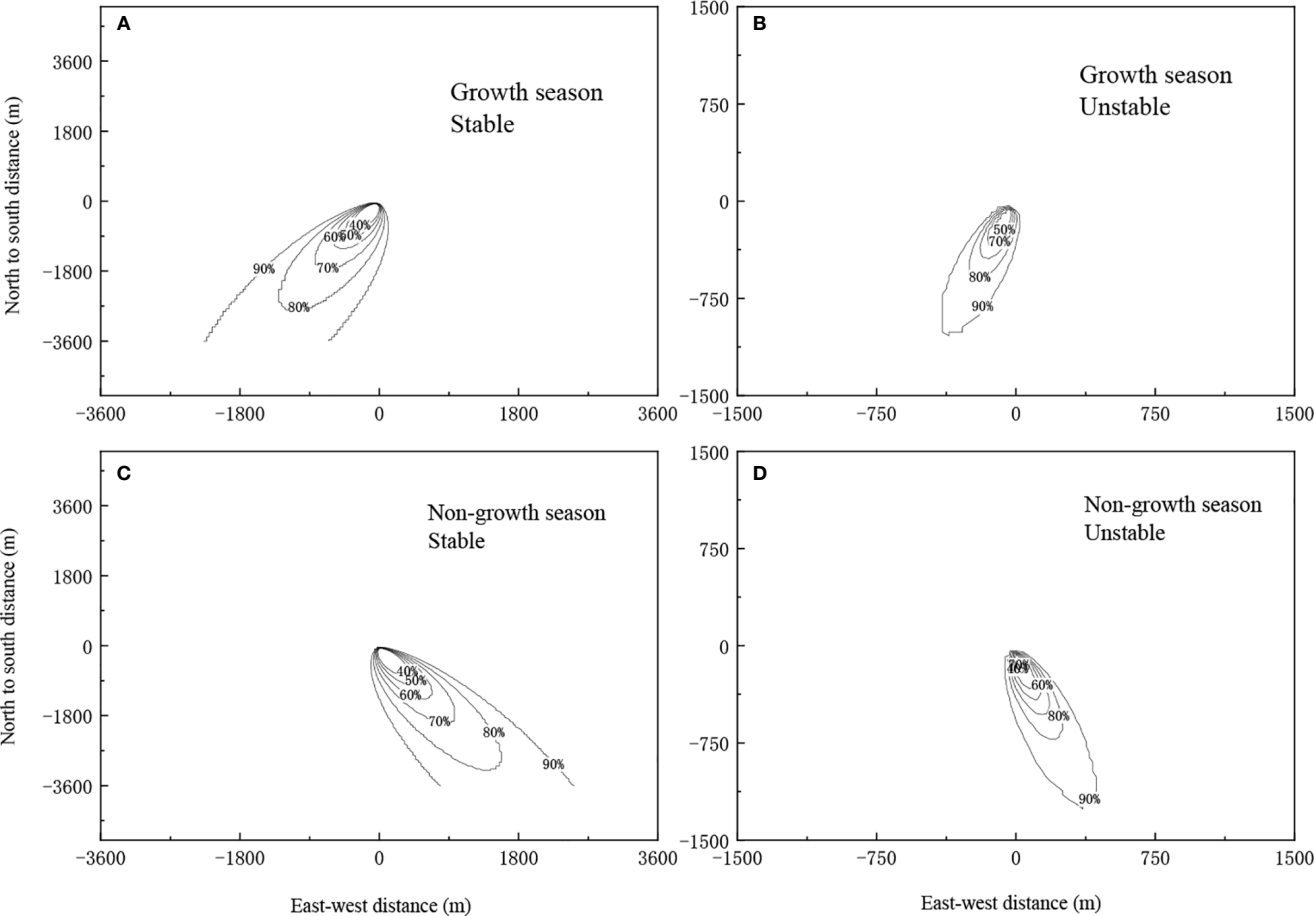
The footprint under different stability conditions during the growth and non-growth seasons of 2017.**(A)** Growth season, Stable. **(B)** Growth season, Unstable. **(C)** Non-growth season, Stable. **(D)** Non-growth season, Unstable.

**Figure 2** shows the flux footprint under different conditions of atmospheric stability during the growth (August) and non-growth seasons (December) in 2017. The footprint was taken 80% as the target, regardless of season (growth or non-growth), the footprint was consistently smaller without atmospheric stability, owing to the intense material exchange between canopy and atmosphere when the atmosphere is unstable. In addition, flux information captured by the sensor mainly came from upwind of the sensor. Moreover, the footprint was smaller during the growth season because the flux measurements were from the underlying surface further to windward of the sensor, as the leaf area index is greater during the growth season and is influenced by the underlying surface.

**Figure 3:**
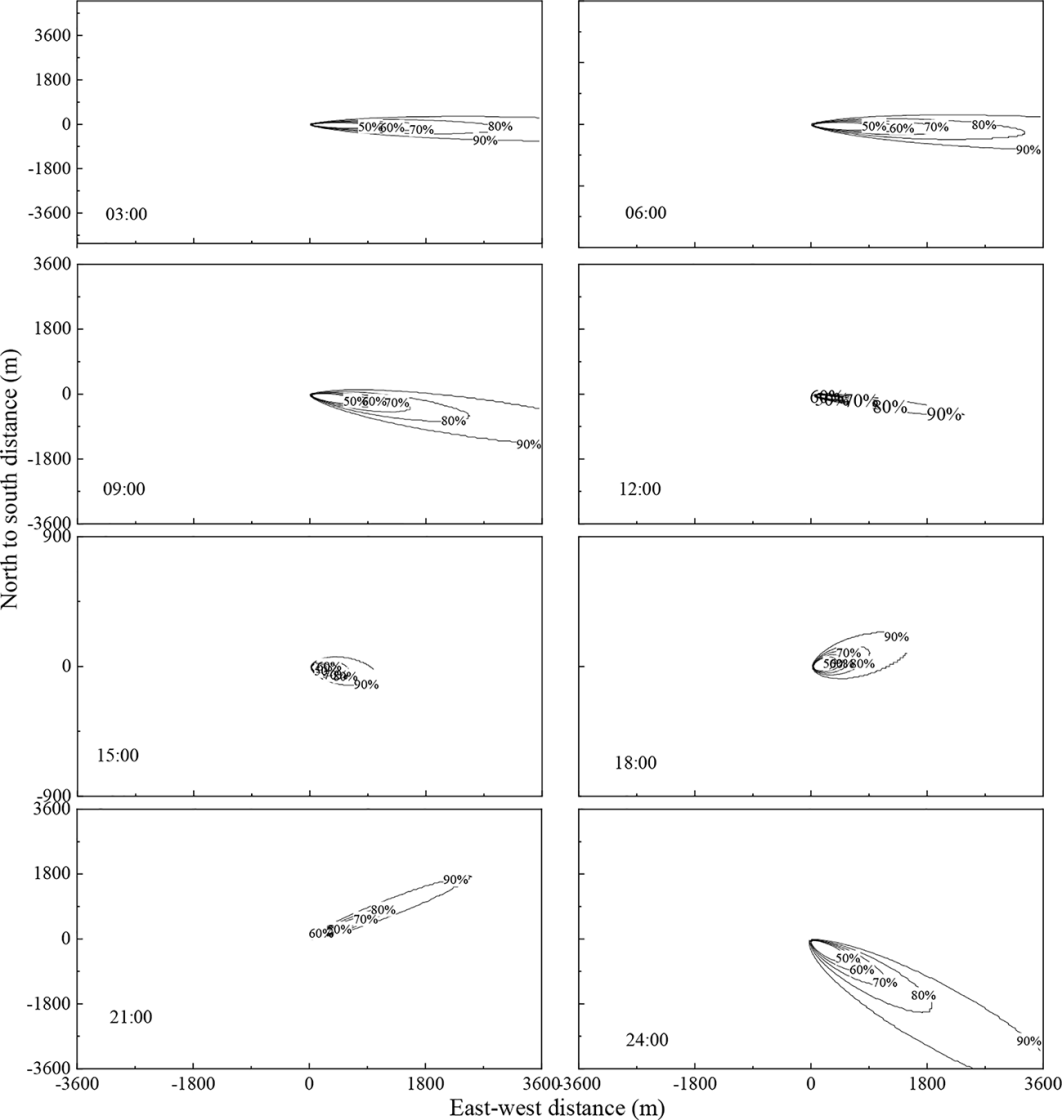
Changes in flux footprint over time within a typical sunny day (August 8^th^, 2017).

The flux footprint throughout August 8^th^, 2017, which was a typical sunny day, was analysed taking 3 h as the time interval. The distribution was non-uniform, and the flux changes throughout the day can be easily observed in **Figure 3**. At 3:00, the flux footprint was approximately 3000 m east to west and 600 m north to south, accounting for an area of 1.26 km^2^; at 9:00, the flux footprint was approximately 2520 m east to west and 720 m north to south, accounting for an area of 1.04 km^2^; at 15:00, the flux footprint was approximately 600 m east to west and 150 m north to south, accounting for an area of 0.06 km^2^; and at 21:00 was approximately 1300 m east to west and 900 m north to south, accounting for an area of 0.28 km^2^. Throughout the day, the flux footprint was in general consistently distributed to windward. In summary, the eddy covariance measurement system measures the size of the windward flux footprint, and the data represented the flux of the study area well.

### 3.2 Characteristics of CH_4_ flux changes with time in ecosystem

#### 3.2.1 Daily change in CH_4_ flux

##### 3.2.1.1 Diurnal variation in CH_4_ flux averaged by month

**Figure 4:**
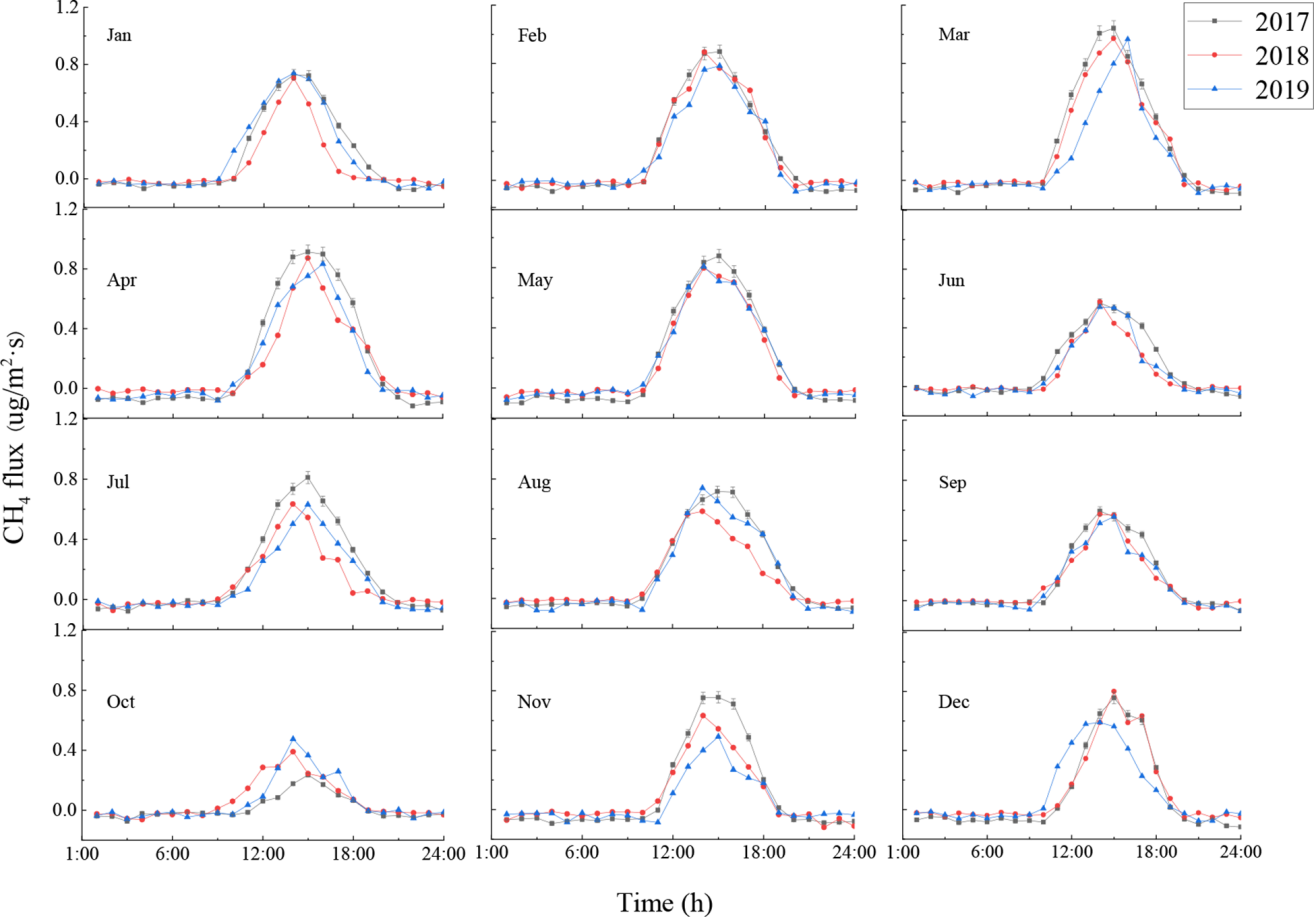
Average within-day CH_4_ flux of the ecosystem by month. Error bars show standard deviation.

The CH_4_ flux in the ecosystem showed obvious changes throughout a day (**Figure 4**). For each month, the average within-day changes in CH_4_ flux followed an inverted U-shape pattern: CH_4_ flux changed from negative to positive after sunrise due to the increase in radiation and temperature, and the ecosystem served as a CH_4_ source in the atmosphere, the CH_4_ flux reached its maximum value at 15:00, after which it decreased gradually as the radiation and temperature decreased. The CH_4_ flux became negative around sunset, causing the ecosystem to become a CH_4_ sink for the atmosphere. The CH_4_ flux remained unchanged during the night, due to the influence of weak turbulence.

The CH_4_ flux of the ecosystem showed similar obvious within-day changes across months, with some months showing slightly different changes (**Figure 4**). The average within-day CH_4_ flux was highest in March, reaching 1.11 μg·m^-2^·s^-1^, 0.97 μg·m^-2^·s^-1^, and 0.99 μg·m^-2^·s^-1^ respectively in 2017, 2018, and 2019. The average within-day CH_4_ flux was lowest in October, reaching 0.26 μg·m^-2^·s^-1^, 0.42 μg·m^-2^·s^-1^, and 0.48 μg·m^-2^·s^-1^ respectively in 2017, 2018, and 2019, which differed markedly from the CH_4_ flux in March. The CH_4_ flux changed from negative to positive at the earliest in July (at around 8:30) and at the latest in November (at around 10:00). The CH_4_ flux changed from positive to negative around July to August at the latest (at around 19:30), and this change gradually became earlier from September until it reached its earliest around 18:00 in December. As a result, the CH_4_ flux remained positive for the longest period in July (11 h), and the shortest period in October (8 h) (**Figure 4**). Radiation and temperature reached their maximum values around June each year in the current study area, yet the CH_4_ flux (both daily maximum and daily average) of the ecosystem was less than in adjacent months, owing to the high temperature and small precipitation in June.

**Figure 5:**
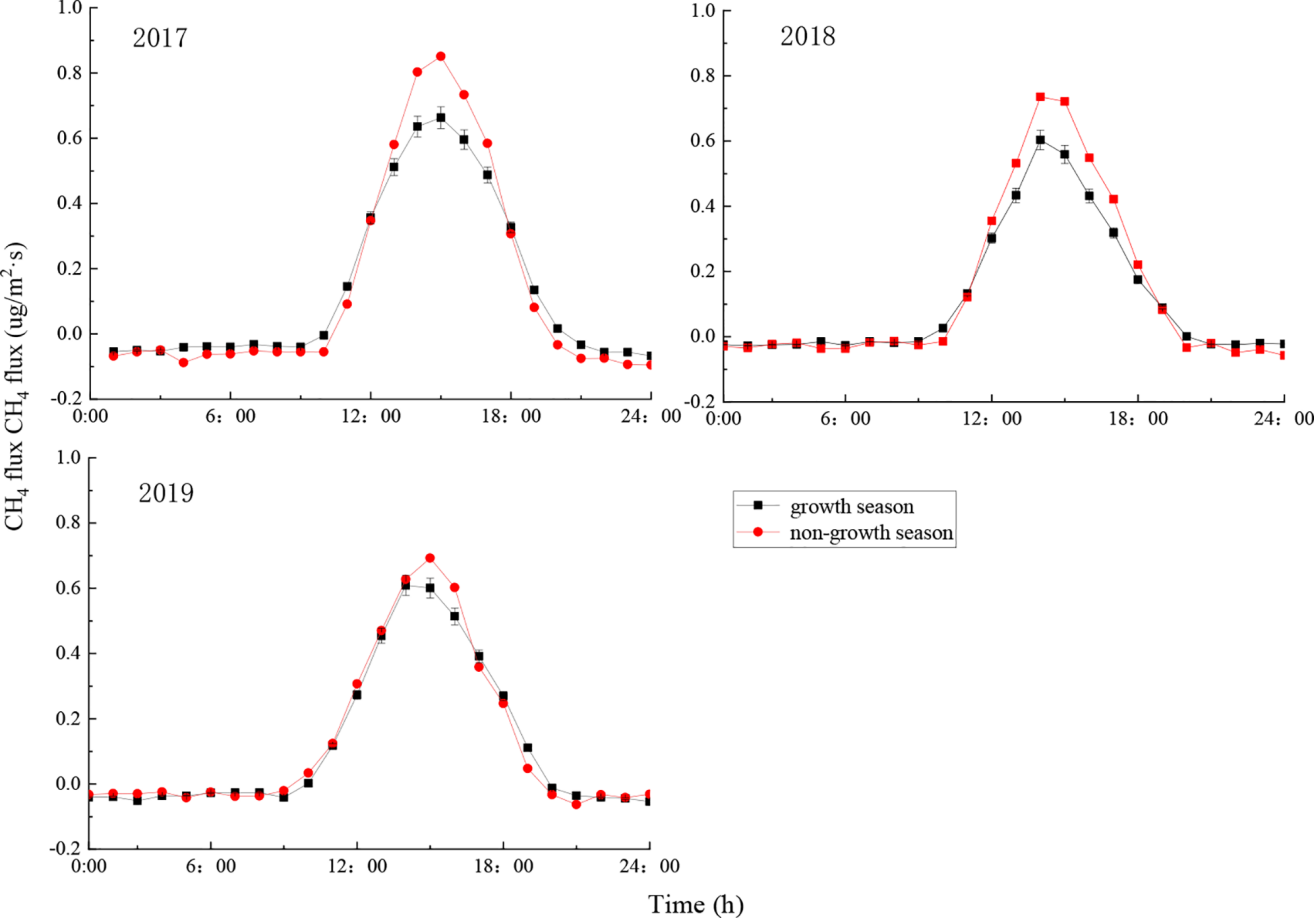
Changes in average within-day CH_4_ flux by month during the growth and non-growth seasons. Error bars show standard deviation.

The CH_4_ flux of the ecosystem during the growth and non-growth seasons during the study period was positive during the day and did not change much during the night (**Figure 5**). During the growth season, the CH_4_ flux increased rapidly from 10:00 to 14:00, and decreased slowly from 14:00 to 20:00. During the non-growth season, the CH_4_ flux also increased rapidly from 10:00 to 14:00 and to a higher peak, and decreased from 14:00 to 20:00 (**Figure 5**). This is consistent with the results reported by Tong et al. (2012) on the CO_2_ flux of the same ecosystem. This can be explained by the relatively high temperature in the afternoon in spring and summer, causing VPD to be relatively large.

The daily maximum of CH_4_ flux was lower during the growth season than during the non-growth season (**Figure 5**), mainly because precipitation is high during the growth season. As the growth season transitions into the non-growth season (November to March), radiation and temperature gradually decrease, and the forest soil serves as a CH_4_ source as most trees, except for coniferous species such as *Platycladus orientalis*, experience withering and leaf fall (Zhuang et al, 2016). Therefore, CH_4_ flux is lower in the growth season.

##### 3.2.1.2 Diurnal variation of CH_4_ flux on sunny and rainy days

**Figure 6:**
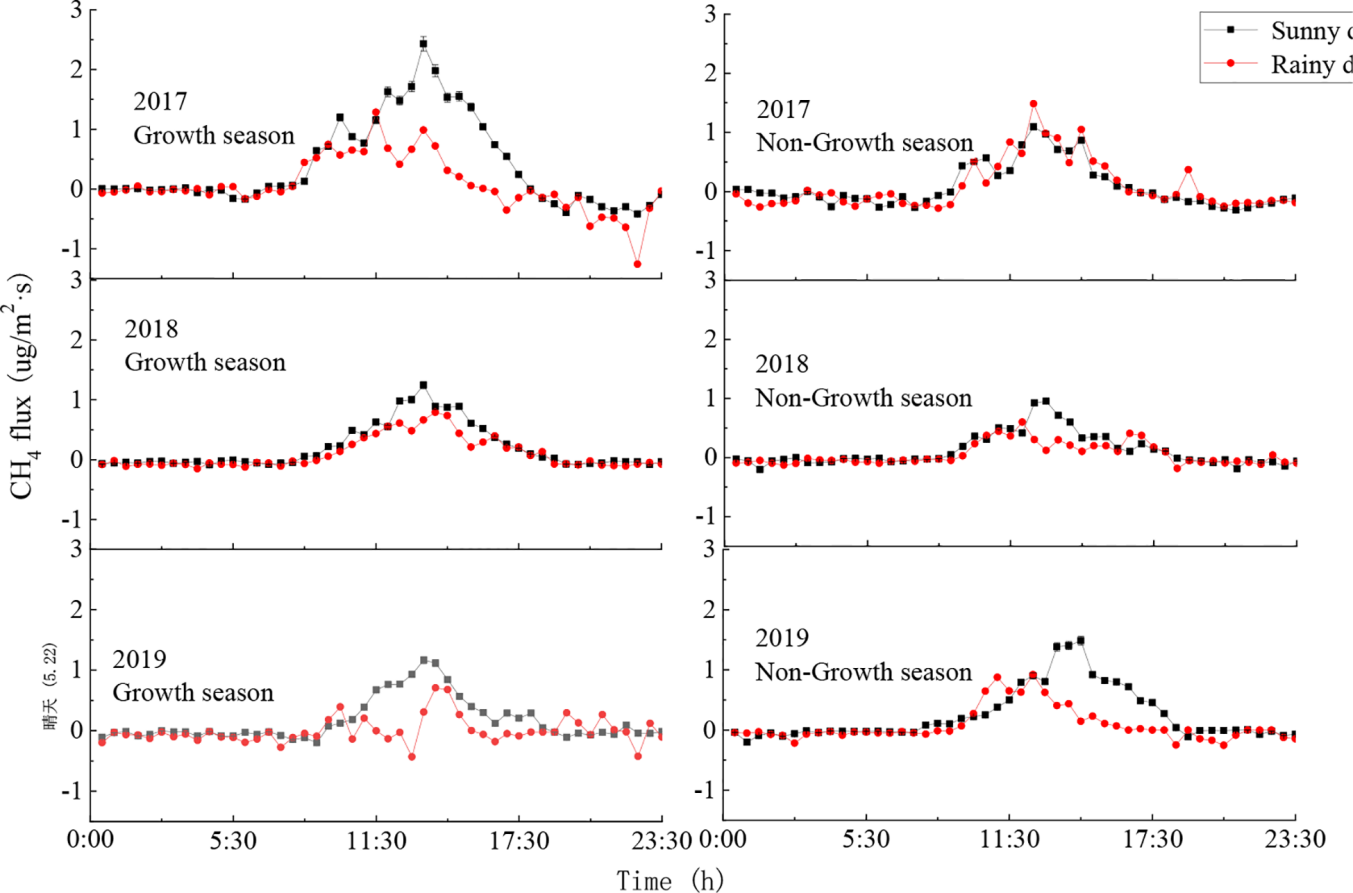
Diurnal variations of CH_4_ flux on sunny and rainy days. Error bars show standard deviation.

Typical sunny days (April 27^th^, 2017; December 6^th^, 2017; February 22^nd^, 2018; September 7^th^, 2018; May 22^nd^, 2019; November 14^th^, 2019) and rainy days (May 22^nd^, 2017; November 28^th^, 2017; February 18^th^, 2018; August 20^th^, 2018; May 29^th^, 2019; November 12^th^, 2019) in the growth and non-growth seasons were selected to analyse the within-day changes in the CH_4_ flux of the ecosystem. These CH_4_ fluxes all followed an inverted U-shape pattern, where the CH_4_ flux was positive during the day and showed significant changes, and the changes were more complicated on rainy days (**Figure 6**). On a sunny day, as radiation and temperature increased after sunrise, CH_4_ flux also gradually increased until reaching its maximum value around 13:30. In contrast, when the atmosphere was relatively stable during the night and the air turbulence was weak, CH_4_ flux showed no significant changes.

During the growth season, CH_4_ flux was positive during the day and showed no significant changes during the night. The daily average CH_4_ fluxes on a typical sunny day in 2017, 2018, and 2019 were respectively 0.38 μg·m^-2^·s^-1^, 0.12 μg·m^-2^·s^-1^, and 0.16 μg·m^-2^·s^-1^, while the daily average CH_4_ fluxes on a typical rainy day were respectively 0.06 μg·m^-2^·s^-1^, 0.05 μg·m^-2^·s^-1^, and 0.27 μg·m^-2^·s^-1^. During the non-growth season, the daily average CH_4_ fluxes on a typical sunny day in 2017, 2018, and 2019 were respectively 0.07 μg·m^-2^·s^-1^, 0.19 μg·m^-2^·s^-1^, and 0.25 μg·m^-2^·s^-1^, while the daily average CH_4_ fluxes on a typical rainy day were respectively 0.08 μg·m^-2^·s^-1^, 0.10 μg·m^-2^·s^-1^, and 0.08 μg·m^-2^·s^-1^. The CH_4_ fluxes were higher on sunny days than on rainy days and reached maximum values in the afternoons on sunny days. In contrast, the CH_4_ flux on a rainy day reached its maximum value before noon.

Typical sunny days showed generally consistent changes in CH_4_ flux throughout the day, while the flux changes on rainy days differed depending on precipitation intensity and amount. During the growth season, the CH_4_ flux on sunny days was slightly higher than on rainy days due to the larger leaf area caused by rainfall. During the non-growth season, both duration and amount of rainfall were reduced, such that CH_4_ flux did not differ significantly between rainy and sunny days. Overall, CH_4_ flux during the day was positive, making the ecosystem a CH_4_ source, and CH_4_ flux at night was negative, making the ecosystem a CH_4_ sink.

##### 3.2.1.3 Diurnal variations of CH_4_ flux during continuous rain

**Figure 7:**
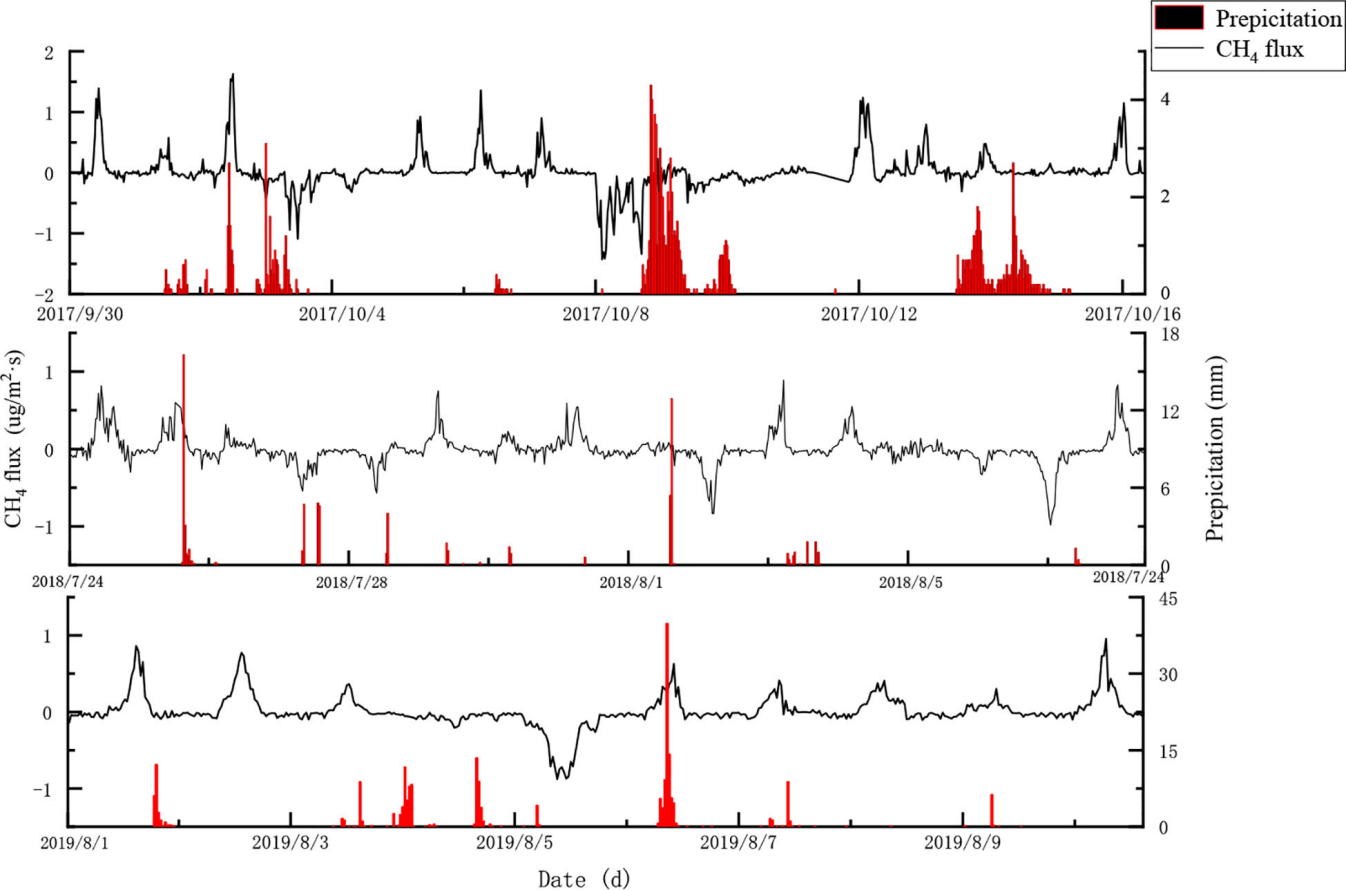
Within-day changes in CH_4_ flux of ecosystem during periods of continuous rainfall.

CH_4_ fluxes before, during, and after periods of continuous rain in 2017, 2018, and 2019 were summarised. The precipitations in these periods were respectively 127.2 mm, 77.7 mm, and 220.5 mm. **Figure 7** shows the diurnal cycle of CH_4_ flux for each continuous rainfall period.

Considering the period before the rain began (to Sep 30^th^) and after rainfall (Oct 16^th^) were sunny days, the diurnal variation in CH_4_ flux was consistent with the forest being a CH_4_ source in the daytime and a CH_4_ sink at night. At the beginning of the continuous rainfall period (Oct 1^st^ to 2^nd^), CH_4_ flux had no change with a precipitation of 12.8 mm; however, the CH_4_ flux exhibited a U-shaped pattern and became a CH_4_ sink during Oct 3^rd^ and 4^th^ (precipitation 13.6 mm). The CH_4_ flux on October 5^th^ to 7^th^ (precipitation 1.6 mm) again changed consistently to become a CH_4_ source. The CH_4_ flux again exhibited a U-shaped pattern on October 8^th^ (precipitation 0.1 mm) and served as a CH_4_ sink, but showed no significant changes from October 9^th^ to 11^th^ (precipitation 62.7 mm). With precipitation 0.1 mm on October 12^th^, CH_4_ flux showed the same pattern as on a sunny day, while during October 13^th^ to 15^th^ it changed a little (precipitation 36.3 mm). The diurnal dynamic of CH_4_ flux showed similarities with June 24^th^ to August 9^th^ in 2018 and August 1^st^ to 10^th^ in 2019 and exhibited an alternation of source/sink.

The continuous rainfall had a significant impact on the within-day changes of CH_4_ flux, through influences on atmospheric temperature, air humidity, soil temperature, and soil humidity. There was a lag of approximately 3 to 4 days between the rainfall and its influence on CH_4_ flux which ultimately led to the source-sink transition of the ecosystem. This means that if the rainfall continued for 3 to 4 days, the ecosystem transformed from a CH_4_ source to a CH_4_ sink. The intensity and duration of the rainfall had a coupled effect on the CH_4_ flux, which further influenced the CH_4_ source-sink transition of the ecosystem.

#### 3.2.2 Seasonal changes in the CH_4_ flux of the ecosystem

**Figure 8:**
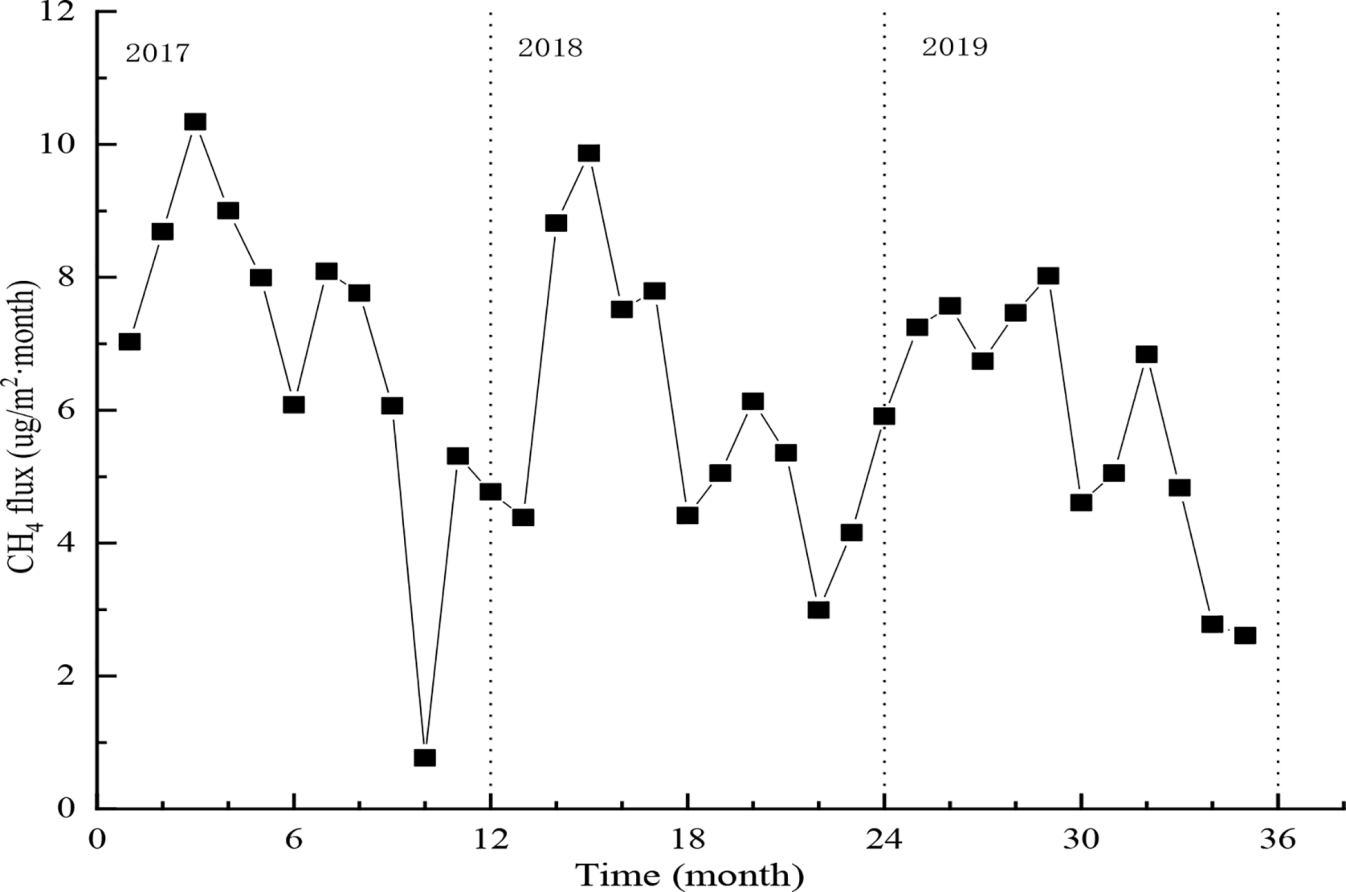
Seasonal changes in CH_4_ flux of ecosystem.

The CH_4_ flux of the ecosystem showed obvious seasonal changes (**Figure 8**). It increased from November to March the following year, and gradually decreased thereafter, reaching a minimum in June, and again increased during July and August, and decreased in September until it reached the year-round minimum in October. These changes are mainly due to the radiation, temperature, precipitation, and vegetation growth during the different months. For example, between January and March in 2017, which is the non-growth season, soil and vegetation branches released CH_4_, causing the forest ecosystem to serve as a CH_4_ source from which the maximum emission of CH_4_ was reached in March (4.47 g·m^-2^·month^-1^). Between April and June in 2017, due to the increase in solar radiation, temperature, and hence leaf area, the CH_4_ flux of the ecosystem gradually reduced, until it reached the first minimum value in June (2.55 g·m^-2^·month^-1^, 1.857 g·m^-2^·month^-1^). July was the start of the rainy season, a period during which the soil moisture increased, causing CH_4_ flux to increase accordingly. It reached a maximum in August (3.5 g·m^-2^·month^-1^). Subsequently, CH_4_ flux decreased as the intensity and frequency of precipitation reduced, until the year-round minimum was reached in October (0.33 g·m^-2^·month^-1^).

The ranges of CH_4_ emissions were 0.008–0.108 g C·m^-2^·month^-1^, 0.031–0.103 g C·m^-2^·month^-1^, and 0.027–0.084 g C·m^-2^·month^-1^ respectively in 2017, 2018, and 2019, with the year-round change in CH_4_ emissions being respectively 3.31 g C·m^-2^·year ^-1^, 2.81 g C·m^-2^·year ^-1^, and 2.94 g C·m^-2^·year^-1^, corresponding to an annual average of 3.02 g C·m^-2^·year^-1^. Total annual emissions of CH_4_ were lowest in 2018, mainly due to the higher precipitation of that year (647.8 mm) in comparison to 2017 and 2019.

## 4 Discussion

According to micro-meteorological theory, CH_4_ flux data taken from locations with a wide underlying surface of flat terrain and uniform canopy can reflect the actual average CH_4_ flux of the ecosystem. However, most observation locations do not have the ideal underlying surface, making it necessary to analyse the spatial representation of the flux observation of the complex underlying surface in the flux data. Therefore, quantitative evaluation of the flux footprint is the basis for a correct understanding of the data; this can be achieved by using the eddy covariance method. An in-depth understanding of the spatial representation of the flux towers and an accurate evaluation of the spatiotemporal distribution of flux footprints can help to obtain a more thorough understanding of the CH_4_ flux sources in the ecosystem.

The flux footprint was heavily influenced by environmental factors such as atmospheric stability, wind speed and direction, atmospheric temperature, underlying surface roughness, and zero plane displacement (Leclerc and Thurtell, 1990; Kljun et al., 2002). In particular, atmospheric stability directly affects the distribution of flux footprint. In this chapter, the results of analysis of wind direction and speed data from 2017 to 2019 were described. These revealed that the study area mainly experienced east and west-south-westerly winds. Regardless of growth or non-growth season, the footprint was smaller when the atmosphere was unstable. This can be explained by the turbulent airflow between the underlying surface and the atmosphere. and the fast exchange of material in the vertical direction, which cause the flux measurements from the windward sites to be greatly affected by the underlying surface. However, the leaf area index is lower during the non-growth season, causing the flux measurements to be taken from sites further downwind, and as a result, the footprint during the non-growth season was larger than that during the growth season. This result is consistent with the results from other ecosystems such as farmland, desert, and grassland (Zhou et al., 2014; Feng et al., 2017; Zhou et al., 2018).

A variety of evidence now makes it clear that all biological surfaces in upland forests have the potential to exchange CH_4_. This included reports of novel sources of CH4 emissions in nominally upland ecosystems, eddy flux evidence of hot spots or hot moments of forest CH_4_ emissions. Clear CH_4_ diurnal variation of the ecosystem exchange showed an inverted U-shape pattern. The CH_4_ flux of the ecosystem after sunrise were a CH_4_ source for the atmosphere due to the increase in radiation and temperature, and reached its maximum around 15:00. While after sunset, the CH_4_ flux showed a CH_4_ sink and had a little change due to the weak turbulence at night. This trend is consistent with the results published by Nakai et al. (2020), but the opposite of what Ueyama et al, 2013 had discovered. Covey and Megonigal (2018) and Pitz and Megonigal (2017) found that tree branches, live or dead alike, were potential CH_4_ sources in montane forests. Machacova et al. (2016) suggested that the branches and stems of mature *Pinus sylvestris* in southern Finland emit CH_4_. The current study found that CH_4_ emissions increased sharply before noon and decreased gradually in the afternoon during spring and summer as the temperature in the afternoon was higher, which is consistent with the results published by Korkiakoski et al., 2017. Without atmospheric stability, CH_4_ flux is related to temperature to a certain extent. In addition, CH_4_ flux during daytime is also dependent on factors such as soil moisture. Precipitation is greater during the growth season, causing the daily average CH_4_ fluxes during the growth season to be greater than those during the non-growth season. The daily average CH_4_ flux was greatest in March and lowest in October. The CH_4_ flux changed from sink to source around 8:30 each day in July, which was the earliest among all months, and around 10:00 in November, which was the latest among all months. The CH_4_ flux changed from source to sink around 19:30 during July and August, which was the latest among all months. This change became earlier starting from September, until the transition took place around 18:00 in December. Consequently, the duration that the CH_4_ flux remained positive was longest in July (11 h) and shortest in October (8 h). This finding is similar to the results published by Querino et al. (2011), who discovered that the duration of positive CH_4_ flux was longer than in a tropical forest ecosystem after sunrise (5 h). Gao et al. (2016) found that positive CH_4_ flux was measured during the daytime during some months in a floodplain plantation ecosystem, which may have been due to the CH_4_ gas stored in the canopy during night-time being released into the atmosphere after sunrise.

The diurnal variations of CH_4_ fluxes in this ecosystem showed obvious trends during the growth and non-growth seasons, being positive during daytime and with non-significant changes at night. This indicates that the CH_4_ flux showed significant changes only when the atmosphere was unstable and the turbulent airflow was strong; at night, when the atmosphere was stable and the turbulent airflow was weak, the CH_4_ flux showed no significant changes. This is consistent with research on night-time CH_4_ flux in farmland and wetland ecosystems (Song et al., 2019; Zhang et al., 2019). The maximum values of diurnal average CH_4_ fluxes during the growth season were all less than those of the non-growth season, mainly because of the larger precipitation and net radiation, the latter of which enhanced plants’ activities and reduced the oxidation of CH_4_ (Praeg et al., 2019). Obvious daily changes were observed during the sunny days in both the growth and the non-growth season. The CH_4_ fluxes were greater on a sunny day than on a rainy day, and the maximum value appeared in the afternoon; in contrast, CH_4_ flux changed in complicated ways on rainy days, when the maximum value appeared before noon, and the daily average was slightly lower. Although soil appears to be a CH_4_ sink during the growth season (Zhuang et al, 2016), the emissions from plants may have offset the effect of the soil CH_4_ sink (Pitz and Megonigal, 2017; LeMer and Roger, 2001).

The comparison of diurnal variations of CH_4_ flux before, during, and after continuous rainfall indicated that the intensity and frequency of rainfall, as well as extreme precipitation events, influenced the CH_4_ source-sink transition. Before and after the rainfall, the CH_4_ flux changed in the same manner as on a typical sunny day. Due to the lag in the influence of rainfall on the CH_4_ flux, the daily patterns of CH_4_ flux only began to change on the third or fourth day of continuous rainfall. They then followed a U-shaped pattern with a negative value during daytime, causing the ecosystem to serve as a CH_4_ sink. As the rainfall continued for more days, the CH_4_ source-sink transition happened approximately every three days. This can be explained by the change in effective dynamic characteristics of soil moisture according to water intake; this being most affected by limited water intake, while the more frequently precipitation events occur, the more dependent soil moisture is on water intake (Zhao et al., 2015).

The CH_4_ flux of this ecosystem showed obvious seasonal changes. As radiation and temperature increased, plants entered the growth season and the leaf area of the canopy increased, causing the CH_4_ flux to gradually increase, reaching a year-round maximum in March. As the leaf area continued to increase with radiation, the CH_4_ flux gradually decreased, until it reached the first minimum in June when the temperature is high and precipitation is low and the physiological activities of the plants can be affected. The rain season started in July or August, causing the soil moisture and hence the CH_4_ flux to increase. Both radiation and temperature decreased after August, and the CH_4_ flux decreased correspondingly. In October, due to the continuous rainfall, the CH_4_ flux reached a year-round minimum. The magnitude of the CH_4_ flux among the seasons was observed in the order of spring > summer > winter > autumn, which is consistent with the results reported by Zona et al. (2013). Zhang et al. (2019) studied the poplar plantations in the Hongze Lake area and found that weak CH_4_ absorption was observed during the growth season, while weak CH_4_ emissions were observed during the non-growth season, causing the ecosystem to serve as a weak CH_4_ sink in a year overall. However, the CH_4_ flux of the ecosystem studied between 2017 and 2019 and reported in this paper had an average of 3.02 g C·m^-2^·year^-1^, indicating that the ecosystem was a weak CH_4_ source, which is inconsistent with the results by Zhang et al. (2019). This inconsistency may be due to the length of study period, climate, and types of trees.

The study revealed that the mixed plantation forest ecosystem in warm temperate continental is methane source, which is consistent with the results of the boreal forests in the United State (Nakai et al., 2020). During the period of 2016 to 2019, the average daily and annual CH_4_ flux was 0.019g C·m^-2^·day^-1^ lower than that of tropical Alan Batu forest, temperate boreal forest ecosystem, subtropical Pinus ponderosa forest and temperate black spruce forest ecosystem (Wong et al., 2018; Nakai et al., 2020; Iwata et al., 2015; Smeets et al., 2009), similar to subtropical plantation ecosystem in China (Gao et al., 2016). It was higher than temperate boreal forest (Ueyama et al., 2013), with the mainly because of the observation methods (**Table 1**). Climate, soil and tree species in different regions are important reasons for CH_4_ fluxes, as well as differences in measurement methods and CH_4_ source intensity or magnitude.

**Table 1:** Mean daily CH_4_ flux measured using the eddy covariance method from different ecosystems in Ameirco, Europe, and Asia.

Ecosystems	Climates	Observation methods	CH_4_ flux (g C m^-2^ day^-1^)	Observation period	References
Alan Batu forest	Tropical	OPEC	0.025	2014.2-2015.7	Wong et al. (2018)
Ponderosa pine	Subtropical	CPEC	0.0018	2007.8.11-19	Smeets et al. (2009)
Poplar plantation	Subtropical	CPEC	0.0029	2012-2013	He et al. (2019);
A mixed plantation	Temperate	CPEC	0.0019	2016.6-2019.11	This study
Boreal forest	Temperate	OPEC	0.036	2014.5.29-6.12	Praeg et al. (2019)
Boreal forest	Temperate	Relaxed eddy accumulation	0.0002	2014-2017	Wilczak et al. (2001)
Black spruce forest	Temperate	CPEC	0.009	2011-2013	Iwata et al. (2015)

## 5 Conclusion

The flux footprint showed non-uniform changes throughout a day and were smaller in the growth season and daytime, reaching a minimum at noon and a maximum at 3 am. The eddy covariance measurement system measures the size of the windward flux footprint, and the data represented the flux of the study area well.

The CH_4_ flux showed obvious patterns in its daily changes. The within-day changes in CH_4_ flux by month followed an inverted U-shape pattern which was source during daytime and a CH_4_ sink at night. The largest daily average CH_4_ flux appeared in March, and the smallest appeared in October.

The CH_4_ flux also showed obvious seasonal changes. The CH_4_ flux reached its maximum in spring, and the first minimum of the year was observed in summer, followed by the year-round minimum in autumn. The flux gradually increased in winter but was still lower than in summer.

Precipitation events affected the CH_4_ source-sink transition with a time lag. The changes in within-day CH_4_ flux began on the third and fourth day of continuous rainfall, and were negative during the daytime (i.e., it was a CH_4_ sink). As rainfall continued, the CH_4_ source-sink transition alternated approximately every three days.

The CH_4_ source-sink status of the ecosystem was relatively complicated. Overall, the ecosystem was a weak CH_4_ source, while the source-sink transition occurred on a daily basis.

Based on the observation data of three years, this paper reports on analyses of the characteristics of changes in the CH_4_ fluxes in the ecosystem. To reduce the uncertainty in evaluating the CH_4_ flux of the ecosystem, continuous observation and measurement are required.

## Data availability statement

The raw data supporting the conclusions of this article will be made available by the authors, without undue reservation.

## Author contributions

WY and HH carried out the data processing and analysis and wrote the manuscript; JZ, PM, JL, and TW contributed to the conception and design of the study; FZ and QP organised the data and performed the statistical analysis. All authors participated in the manuscript editing and approved the final version.
